# Deciphering dermatofibromas: A confocal and dermoscopic perspective for enhanced diagnostic precision

**DOI:** 10.1016/j.jdcr.2024.05.031

**Published:** 2024-06-05

**Authors:** Hailey Konisky, Krishna Sharma, Aashka Suvarnakar, Neal Gregory, Albert Huho

**Affiliations:** a Albert Einstein College of Medicine, Bronx, New York; b Georgetown University, Washington, District of Columbia; c Upstate Dermatology, Clinical and Mohs Services, Castleton-on-Hudson, New York

**Keywords:** dermatofibroma, dermoscopy, histology, reflectance confocal microscopy

## Introduction

Dermatofibromas are common benign skin lesions presenting as single, firm, subcutaneous nodules, often occurring at sites of prior trauma. Given their firm nature, a “dimple sign” is typically elicited when squeezing the skin surrounding the dermatofibroma. Though dermatofibromas are benign, the differential diagnosis includes dermatofibrosarcoma protuberans, Kaposi sarcoma, and basal cell carcinoma. Therefore, biopsies of dermatofibromas may occasionally be required.^1^ Reflectance confocal microscopy (RCM) is a noninvasive *in vivo* imaging of the skin that has gained traction in dermatology clinical practice for use in detecting and monitoring skin cancers. Recently, RCM has been utilized for imaging a wider variety of skin lesions, including dermatofibromas.^2^ Dermatofibromas may present a challenge to confocal microscopists when clinical history lacks explicit mention of “dimple sign”. Herein, we describe the dermoscopic and RCM features that may help more confidently diagnose dermatofibromas in the absence of adequate clinical description. This study also attempts to describe in further detail the correlation between dermoscopic, RCM, and histologic features of dermatofibromas.

## Methods

Cases diagnosed as dermatofibromas over the last 4 years were identified in the RCM archives of Upstate Dermatology, a private outpatient clinic in Upstate, New York. Cases selected for further detailed analysis either had biopsy confirmation or documentation of a clear dimple sign at the bedside by a clinician. Dermoscopy, RCM, and histological findings were systematically reviewed and analyzed to reveal the most frequent features and reinforce pattern recognition.

## Results

**Fig 1:**
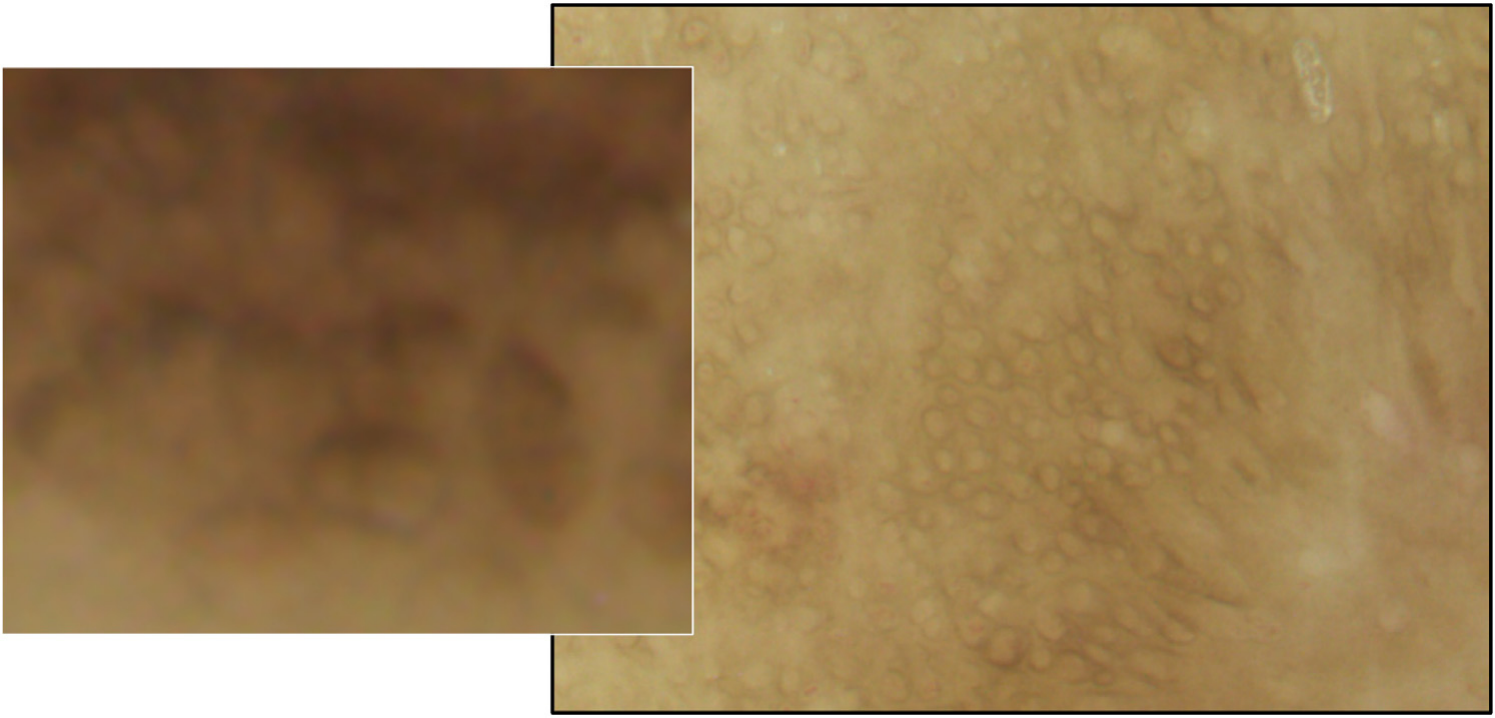
Dermoscopy: Polarized asymmetrically pigmented rings as part of a pigment network, in a dermatofibroma. Note some rings appear separate from the network and the pigment is asymmetrically distributed around the ring with polarization towards 1 side. The *left* image is a zoomed-in view of the *right* image.

**Fig 2:**
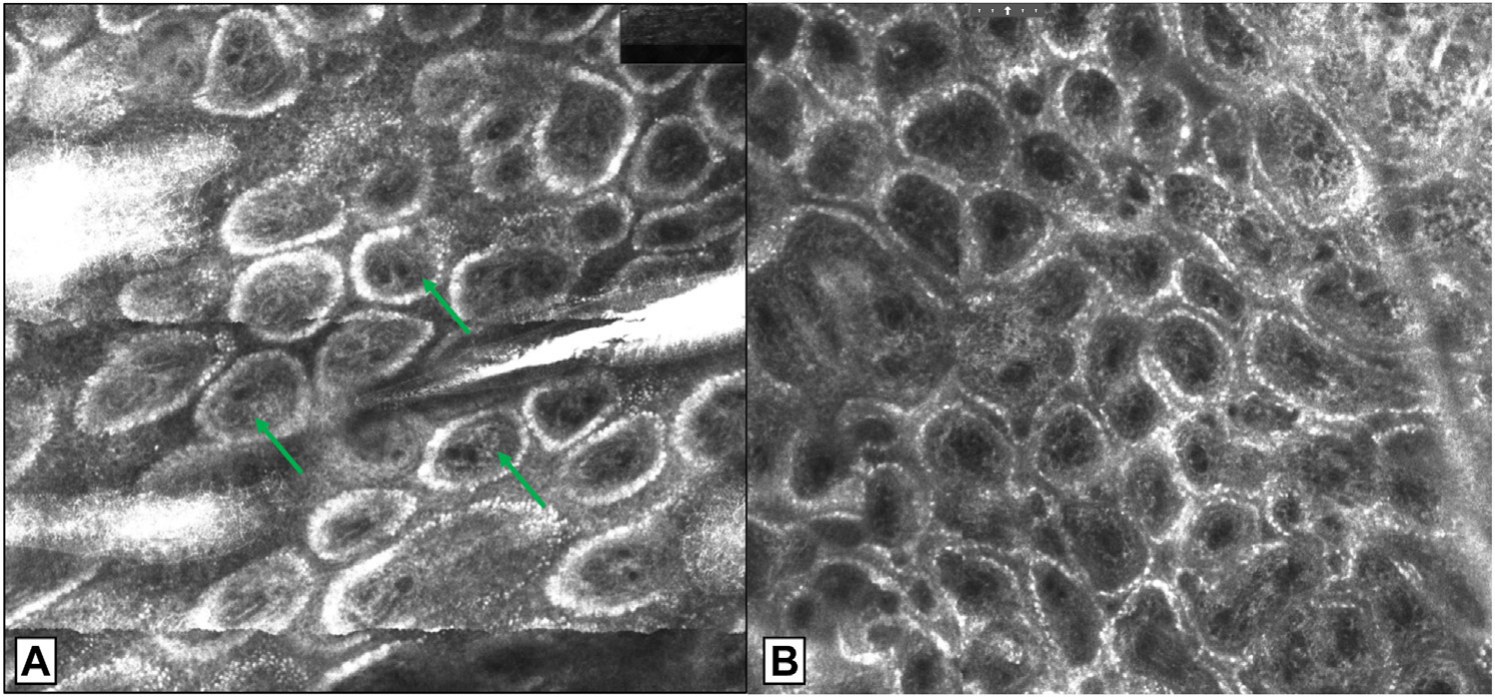
Reflectance confocal microscopy: **A,** Asymmetrically refractive dermal papillae (rings on dermoscopy). Note the fibrosis (*greyish* areas) surrounding the central papillary dermal vessels (*green arrows*) giving a double contour appearance to the rings. **B,** Surrounding normal skin is a good control to help contrast and identify dermal fibrosis.

**Fig 3:**
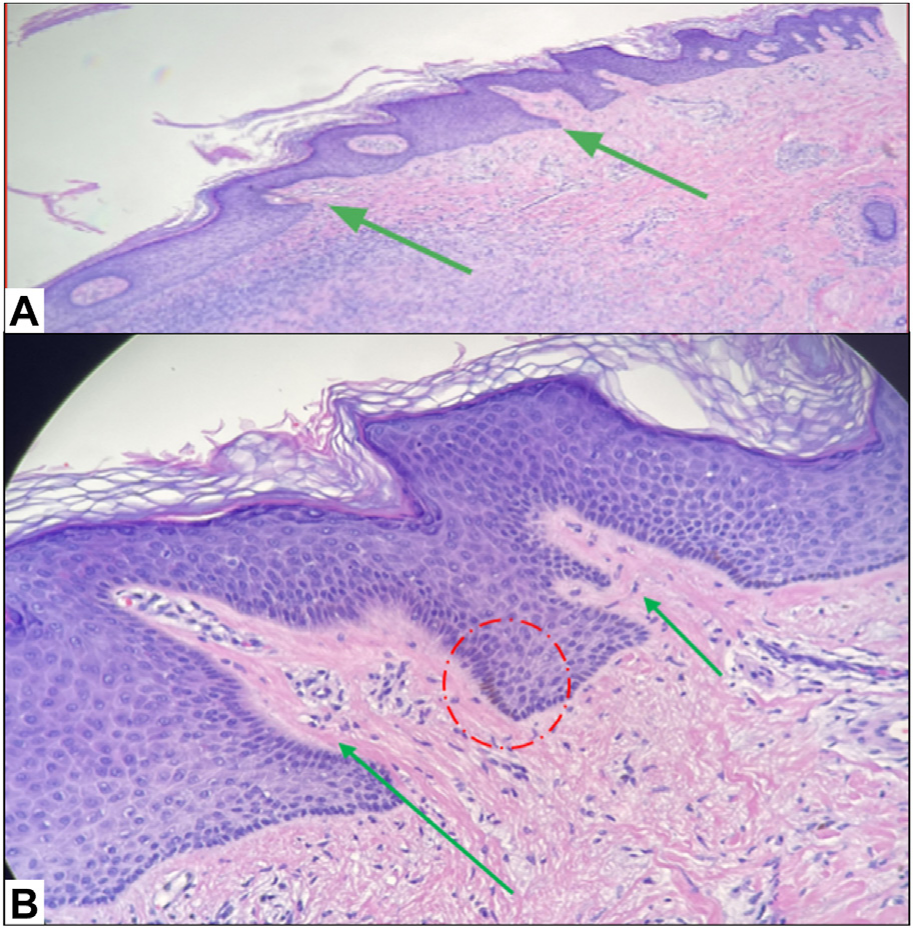
Histology: **A,** Histology of dermatofibroma showing epidermal hyperplasia with tilted papillae giving a “saw tooth-like” configuration (*green arrows*). **B,** Higher power shows subtle fibrosis extending from the dermis into the dermal papillae (*green arrows*). Please note basilar hyperpigmentation (*red circle*).

Twenty-nine cases were identified (3 cases were biopsy-confirmed and 26 cases were retrospectively described as “typical” for dermatofibromas). Seventy-five percent (22/29) of cases had a typical dermatofibroma appearance on digital dermoscopy (ie polarized asymmetrically pigmented round to ovoid well-defined ring-like structures centrally (Fig 1).^3^ This pattern correlated with well-defined round rings with asymmetric reflectance on RCM, which was noted in 90% of our patients (Fig 2). One hundred percent of patients exhibited well-defined rings with papillary dermal fibrosis giving a double contour appearance on RCM (Fig 2). This finding correlated histopathologically with papillary dermal fibrosis (Fig 3). Dermatofibromas also typically exhibited well-defined epidermal hyperplasia with a “saw tooth-like” pattern with basilar hyperpigmentation on histology (Fig 3).

## Discussion

Dermoscopy and RCM findings evaluated together can enhance diagnostic confidence in dermatofibromas. Asymmetrically pigmented but well-defined rings, whether observed on dermoscopy or RCM, in combination with "double contour" dermal papillae seen on RCM was characteristic of dermatofibromas in our study. This contrasts with a recent study of 40 patients in which only 12.5% of dermatofibromas exhibited “double ring” dermal papillae.^4^ Our study also saw a higher percentage of “streaming pattern” of keratinocytes (86% vs 30%). The most common RCM features noted in the other study were a honeycombed appearance of the epidermis (100%), and edged papillae and single rings in the dermis (100%).^4^ More prospective and larger studies are needed to validate the diagnostic utility of these findings in dermatofibromas.

## Conflicts of interest

None declared.
